# A Rare Case of Thyroid Metastasis From Colorectal Cancer: Diagnostic and Therapeutic Challenges

**DOI:** 10.7759/cureus.50097

**Published:** 2023-12-07

**Authors:** Dimitra Ioanna Lampropoulou, Evangelia Pliakou, Maria Ourania Panagiotou, Theodoros Mariolis-Sapsakos, Gerasimos Aravantinos

**Affiliations:** 1 Clinical Research, Econcare, Athens, GRC; 2 Oncology, General Oncology Hospital "Agioi Anargyroi" in Kifissia, Athens, GRC; 3 Nursing, National and Kapodistrian University of Athens, Athens, GRC; 4 Oncology, Athens Euroclinic, Athens, GRC

**Keywords:** rare metastases, thyroidectomy, thyroid mass, thyroid metastases, colorectal cancer

## Abstract

The incidence of thyroid metastases among patients suffering from primary colorectal cancer is rare, and only a few cases have been described in the literature. As these metastases are usually asymptomatic, they most frequently present as incidentalomas on follow-up imaging. Hereby, we present and discuss an interesting case of metastatic sigmoid adenocarcinoma of the thyroid gland, diagnosed and treated at our institution.

## Introduction

The incidence of thyroid metastasis from all tumor types ranges between 1.2% and 24%, with the most common primary sites being the lungs, kidneys, and breast, followed by gastrointestinal neoplasms, melanomas, and lymphomas [1,2]. This low incidence is attributed to the rich and fast arterial flow through the gland, as well as to high values of oxygen and iodine, which would prevent the adhesion and growth of tumor cells [3,4]. Additionally, the relatively short overall survival of patients with advanced malignancies restrains the assessment of newly detected thyroid metastases. To date, less than 120 cases of metastatic colorectal adenocarcinomas of the thyroid gland have been reported [5,6]. Herein, the case of a patient with colon cancer that metastasized to the thyroid gland is presented.

## Case presentation

A 45-year-old female smoker with congenital solitary kidney and no other significant comorbidity was diagnosed with a de novo metastatic adenocarcinoma of the sigmoid colon (KRAS mutated). She received first-line chemotherapy with FOLFOX6 (5‐FU, leucovorin, oxaliplatin), resulting in complete remission of the pulmonary metastases. Subsequently, she underwent rectosigmoidectomy (yT2N0), followed by chemotherapy. After receiving eight cycles with FOLFOX6, pulmonary recurrence was detected, and a left lower lobectomy was performed in 2015. Given the patient’s refusal to receive any further treatment, a close follow-up assessment (every three months) was decided. The imaging findings of the first evaluation revealed the presence of gradually increasing pulmonary nodules (d=2-3 mm). Metastasectomy or cyberknife were suggested, both of which were rejected by the patient. Therefore, she was subjected to chemotherapy with bevacizumab-FOLFOX for 12 cycles, followed by bevacizumab-capecitabine maintenance, achieving stable disease for 11 months.

In 2018, during her follow-up visit, she presented with a firm, rapidly growing (since about two months) thyroid mass causing neck discomfort, without hoarseness, dysphagia, or any apparent cervical adenopathy. The patient was euthyroid (all thyroid tests were within normal limits), and she had no history of previous thyroid disease. Increasing serum CEA and CA 19-9 levels were also detected.

**Figure 1:**
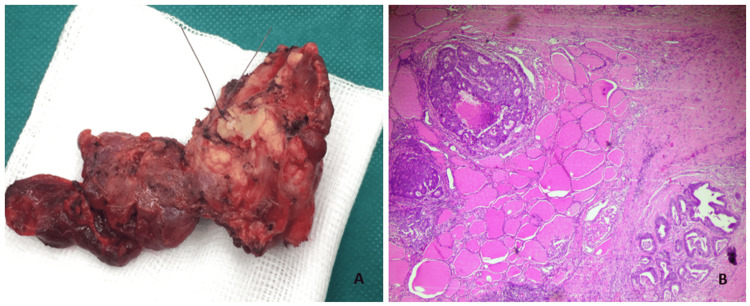
(A) Gross image of thyroid gland removed with partial resection of the thyroid cartilage of the larynx. (B) Diffuse infiltration of the thyroid gland by cribriform nests of neoplastic cells with accompanied necrosis in hematoxylin and eosin stain (x400). The histologic features were consistent with the diagnosis of thyroid metastasis from a primary colorectal neoplasm.

The ultrasound confirmed the presence of a solid, hypoechoic thyroid nodule with ill-defined margins, about 50 mm in size, in the right lobe. An ultrasound-guided fine-needle aspiration (FNA) of the thyroid nodule was obtained, and cytology revealed malignant cells. A total thyroidectomy with partial resection of the thyroid cartilage of the larynx (as it was also occupied by the mass) was performed without any post-operative complications. A gross examination of the thyroid showed complete occupancy of the right lobe by a neoplastic tumor, with a maximum diameter of 5 cm (Figure 1A). The histopathological report diagnosed diffuse infiltration of the right lobe of the thyroid gland by the known sigmoid adenocarcinoma (Figure 1B).

**Figure 2:**
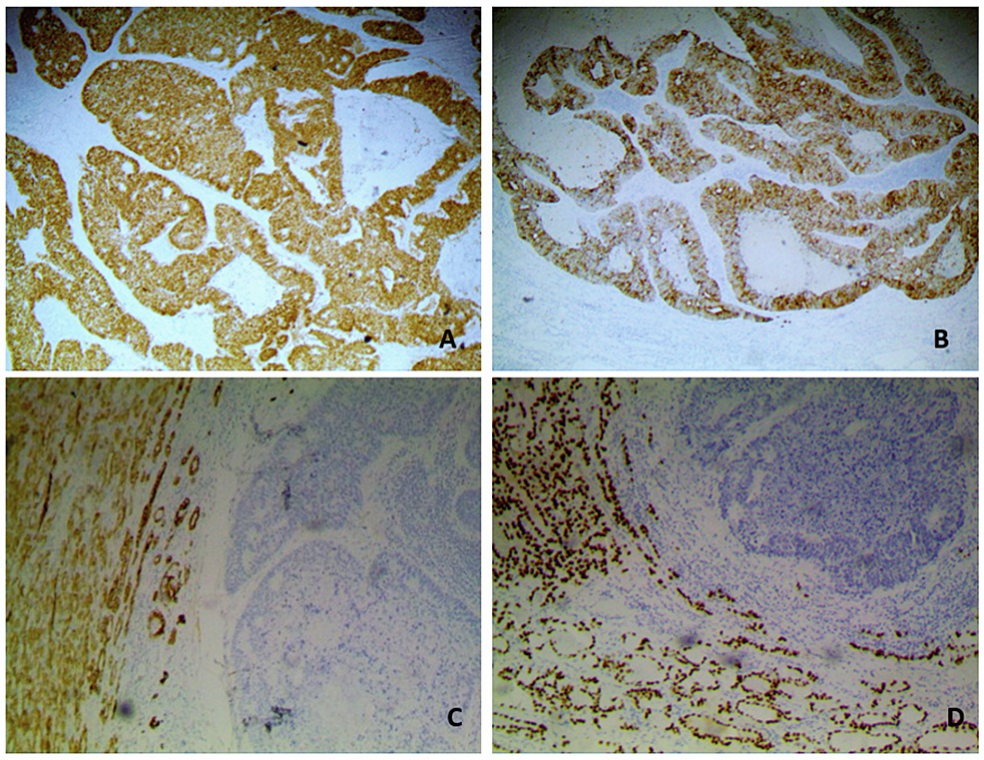
(A) Immunohistochemistry for CDX2 (x400): intense nuclear staining of the neoplastic cells. (B) Immunohistochemistry for CK20 (x400): strong cytoplasmic and membranous immunostaining to CK20 of the metastatic colonic carcinoma. (C) Immunohistochemistry for CK7 (x100): negative cytokeratin expression of the tumor on the right, in contrast to the surrounding thyroid parenchyma. (D) Immunohistochemistry for TTF-1: negative immunoreactivity of the metastatic tumor cells. In contrast, dense and diffuse nuclear staining is observed in the surrounding follicular cells of the thyroid parenchyma.

Moreover, the immunohistochemical staining results were positive for the gastrointestinal markers CDX2 and CK20, whereas negative for CK7 and thyroid-transcription-factor 1 (TTF-1) (Figure 2).

## Discussion

Thyroid metastases of extra-thyroid origin are an uncommon finding that seems to appear metachronously to the primary tumor in most cases, presenting histological rather than clinical findings [7]. The most common primary tumors reported in clinical series to metastasize to the thyroid include renal cell carcinoma (25%) and lung cancer (22%), followed by carcinoma of the breast and gastrointestinal tract (13%) [1,8].

A literature review of metachronous colon metastases to the thyroid gland by Froylich et al. identified only 34 cases from 1931 to 2013 originating from the following sites: rectum (41%), sigmoid colon (33%), ascending colon (19%), and descending colon (11%) [9]; since the review in 2013, Hussein et al. reported 14 cases describing thyroid metastases of colorectal origin [6]. What is more, in a comprehensive systematic review by Manatakis et al., 111 patients were identified to have colorectal thyroid metastasis [5].

The mean age of patients was 61 years in almost all retrospective series on thyroid metastases [8,10]. Interestingly, the majority of the cases in most retrospective series concerned female patients indicating hormonal involvement (female-to-male ratios between 1.1 and 1.5) [8,11-12].

The time interval for thyroid metastatic recurrence varied from six months to eight years after the surgical resection of the primary tumor [9]. In our case, the neoplasm of origin was stage IV sigmoid colon, and thyroid metastasis was detected four years and five months after the diagnosis of the primary tumor.

Patients with thyroid metastases can be asymptomatic or present with goiter-related symptoms, such as the presence of a neck mass, neck edema, dysphagia, and hoarseness [2]. It is noteworthy that the majority of thyroid metastases (72-75%) are symptomatic, like in our patient, which is in agreement with the existing literature [13].

Thyroid masses can be evaluated by several imaging methods. Of note, the use of PET scans has been recommended in both the initial and follow-up staging of patients with colorectal primary neoplasms, as increased thyroidal 18-FDG uptake may suggest the presence of thyroid metastasis [14]. Nevertheless, FNA usually establishes the diagnosis, but it should be noted that it has a low specificity in some cases [15]; also, it is often challenging to differentiate between metastasis and a primary poorly differentiated thyroid cancer [2]. After all, the diagnostic accuracy seems to be affected by the type of malignancy. Metastatic adenocarcinomas of breast, lung, and colorectal origin are often correctly diagnosed, whereas metastatic squamous cell carcinomas of oesophageal and cervical origin can be challenging to diagnose [6]. FNA accurately diagnosed colorectal cancer thyroid metastases in our case.

Immunohistochemical staining could be the key to the distinction; in general, primary thyroid tumors are positive for the thyroid-specific markers TTF-1, thyroglobulin, and the cellular keratin CK7 and negative for CK20. On the other hand, in cases of metastatic colon cancer, the results are almost always positive for CK20 and negative for CK7 [15-18]. In line with the literature data, the immunohistochemical staining results of our patient were positive for the gastrointestinal marker CK20 and negative for CK7 and TTF-1.

The most common treatment strategy is surgical excision, which will prevent the development of uncomfortable symptoms such as dysphagia or imminent airway obstruction [19]. Prophylactic regional lymph node dissection is not generally suggested due to the rarity of neck lymphatic involvement [1,16]. Recent reports recommend the use of chemotherapy alone or in combination with surgery and/or radiation therapy as a good treatment option for patients not amenable to surgery [16]. The surgical strategy should therefore be individualized, ideally in the setting of a multidisciplinary team [1].

In our case, the patient underwent total thyroidectomy with negative post-operative margins without prophylactic neck lymph node dissection.

## Conclusions

The prevalence of metastasis in the thyroid gland from primary colorectal cancer may be underestimated, as it represents a rare clinical entity and requires a low threshold of suspicion. The diagnosis becomes even more challenging since the patients are usually asymptomatic with normal endocrine profiles. The mainstay of treatment is surgical excision, but the decision regarding the final treatment strategy depends on the extent of thyroid metastasis and overall disease, the patient's general condition, and the presence of local symptomatology.

## References

[1] Nixon IJ, Coca-Pelaz A, Kaleva AI (2017). Metastasis to the thyroid gland: a critical review. Ann Surg Oncol.

[2] Ciriano Hernández P, Martínez Pinedo C, Calcerrada Alises E (2020). Colorectal cancer metastases to the thyroid gland: a case report. World J Gastrointest Surg.

[3] Willis RA (1931). Metastatic tumours in the thyreoid gland. Am J Pathol.

[4] Rösner H, Möller W, Groebner S, Torremante P (2016). Antiproliferative/cytotoxic effects of molecular iodine, povidone-iodine and Lugol's solution in different human carcinoma cell lines. Oncol Lett.

[5] Manatakis DK, Tasis N, Antonopoulou MI, Kordelas A, Balalis D, Korkolis DP, Tseleni-Balafouta S (2021). Colorectal cancer metastases to the thyroid gland-a systematic review : colorectal cancer thyroid metastases. Hormones (Athens).

[6] Hussain SM, Cole S, Hussain I (2023). Colorectal cancer metastases in thyroid: case report and literature review. Thyroid Res.

[7] Zivaljevic V, Jovanovic M, Perunicic V, Paunovic I (2018). Surgical treatment of metastasis to the thyroid gland: a single center experience and literature review. Hippokratia.

[8] Chung AY, Tran TB, Brumund KT, Weisman RA, Bouvet M (2012). Metastases to the thyroid: a review of the literature from the last decade. Thyroid.

[9] Froylich D, Shiloni E, Hazzan D (2013). Metachronous colon metastasis to the thyroid: a case report and literature review. Case Rep Surg.

[10] Keranmu A, Zheng H, Wu Y (2017). Comparative study of single-center patients with thyroid metastases from colorectal cancer and previously reported cases in the literature. World J Surg Oncol.

[11] Hegerova L, Griebeler ML, Reynolds JP, Henry MR, Gharib H (2015). Metastasis to the thyroid gland: report of a large series from the Mayo Clinic. Am J Clin Oncol.

[12] Yamamoto T, Tanaka S, Nakamura Y (2012). Colorectal cancer metastasis to the thyroid. Osaka City Med J.

[13] Montero PH, Ibrahimpasic T, Nixon IJ, Shaha AR (2014). Thyroid metastasectomy. J Surg Oncol.

[14] Malani AK, Gupta C, Rangineni S, Gupta V (2005). Thyroid metastasis from colorectal cancer: role of [18F]-fluoro-2-deoxy-D-glucose positron emission tomography. Clin Colorectal Cancer.

[15] Wood K, Vini L, Harmer C (2004). Metastases to the thyroid gland: the Royal Marsden experience. Eur J Surg Oncol.

[16] Kumamoto K, Utsumi Y, Sugano K, Hoshino M, Suzuki S, Takenoshita S (2006). Colon carcinoma metastasis to the thyroid gland: report of a case with a review of the literature. Tumori.

[17] Minami S, Inoue K, Irie J (2016). Metastasis of colon cancer to the thyroid and cervical lymph nodes: a case report. Surg Case Rep.

[18] Melis C, Ballaux F, Bourgain C (2018). Curious residents of the thyroid gland: two case reports of colorectal carcinoma metastasis by fine-needle aspiration diagnosis. Acta Cytol.

[19] Nixon IJ, Whitcher M, Glick J (2011). Surgical management of metastases to the thyroid gland. Ann Surg Oncol.

